# The evaluation of next-generation probiotics on broiler growth performance, gut morphology, gut microbiome, nutrient digestibility, in addition to enzyme production of *Bacillus* spp. in vitro

**DOI:** 10.1016/j.aninu.2024.03.013

**Published:** 2024-04-06

**Authors:** Jacoba I. Bromfield, Shahram Niknafs, Xiaojing Chen, Juhani von Hellens, Darwin Horyanto, Baode Sun, Lei Yu, Viet Hai Tran, Marta Navarro, Eugeni Roura

**Affiliations:** a Bioproton Pty Ltd, Acacia Ridge, Brisbane, Queensland, Australia; b Centre for Nutrition and Food Sciences, Queensland Alliance for Agriculture and Food Innovation, The University of Queensland, Brisbane, Queensland, Australia; c Centre for Animal Science, Queensland Alliance for Agriculture and Food Innovation, The University of Queensland, Brisbane, Queensland, Australia

**Keywords:** Broiler, Probiotic, *Bacillus*, Gut health, Microbial profile

## Abstract

Considerable research has been conducted into the efficacy of individual probiotics in broiler production, however information on the most effective combinations of synergistic *Bacillus* probiotic is lacking. This study investigated the impact of different *Bacillus* strain combinations in broiler chickens, as well as in vitro enzyme production. In experiment one, a total of 576 Ross 308 broilers at 1 d old were grown for 21 d across 6 treatments of maize-soybean diets (*n* = 12 pens per treatment) to compare three different strain combinations (formulation 1 [F1]: 3 strains *Bacillus amyloliquefaciens*; F2: *Bacillus coagulans* and 2 strains *B. amyloliquefaciens*; F3: *B. coagulans, Bacillus licheniformis* and 2 strains *B. amyloliquefaciens*; F5: *Bacillus subtilis, B. licheniformis* and 2 strains *B. amyloliquefaciens*), positive control (PC), and a negative control antibiotic treatment group (NC). In Exp. 2, a total of 360 one-day-old ROSS308 broilers were used to test five treatments (*n* = 9) including PC, NC, F1 and F5 (selected from Exp. 1), and F4 (*Bacillus pumilis* and 2 strains *B. amyloliquefaciens*) in a maize-soybean diet. *B. amyloliquefaciens* F1 demonstrated a significant improvement in feed conversion ratio (FCR) compared to F2 at d 14 (1.49 vs 2.10; *P* = 0.038) and the body weight (BW) at d 21 (847.0 g vs 787.4 g) compared to other combinations (*P* = 0.027). The FCR at d 21 tended to be lower in birds fed F1 (1.46 vs 1.66) compared to the control (*P* = 0.068). Probiotic treatments had significantly improved nutrient digestibility compared to the PC and NC. Also, probiotic treatments supported the growth of *Streptococcus*, a common commensal genus and reduced the abundance of genera that correlated with low weight gain such as *Akkermansia*. Experiment two revealed that F4 improved FCR (*P* < 0.001) and BW at 28 d (*P* = 0.014). In vitro testing showed a high production of protease and amylase by *Bacillus*. Thus, the addition of *Bacillus* probiotics, particularly containing *B. amyloliquefaciens* strains and *Bacillus pumilus*, into the diet of broiler chickens improves production performance, nutrient digestibility, and allows the proliferation of beneficial gut microbiota.

## 1 Introduction

Antibiotics have been historically used in subtherapeutic doses in livestock diets to improve feed conversion ratio and decrease mortality rate, also referred to as antibiotic growth promoters (AGP). In recent times the use of AGP have been banned in many countries due to the increasing levels of antimicrobial resistance (AMR) globally (Salim et al., 2018). However, there are still projections that in certain regions, particularly in Asia, the use of AGP will increase 11.3% by 2030 (Tiseo et al., 2020). This is of great concern as subtherapeutic dosing of livestock presents a significant risk to animals and humans in terms of AMR (Magnusson et al., 2021). Therefore, it is imperative to provide effective alternatives to AGP for utilisation in livestock industries that can be easily adopted at a global scale.

Probiotics are one possible alternative for AGP. A probiotic is a live microorganism that confers health benefits to the host on application (Hill et al., 2014). Historically, probiotics have been used for human health for centuries in the form of fermented food, however this was not implemented in the livestock industry until the 1970s (Gasbarrini et al., 2016; Yirga, 2015). Since this implementation, the probiotic industry has steadily become one of the most lucrative feed additive markets, with multibillion dollars' worth recorded in 2020, and an expected compound annual growth rate of 8.4% by 2031 (Ramlucken et al., 2020).

Spore-based *Bacillus* probiotics, such as *Bacillus subtilis*, are becoming increasingly popular due to their high environmental tolerance, easy storage, and ability to produce other beneficial by-product compounds such as lantibiotics (a group of peptides with antimicrobial activity) and enzymes (Saggese et al., 2021; Wang et al., 2015). Typically, there are 4 primary modes of actions exhibited by probiotics: competitive exclusion, improvement in digestion and absorption, immunomodulatory effects, and a reduction of toxic compounds (Ramlucken et al., 2020). The application of multi-strain probiotics has increased substantially in the last 5 years to capitalise on the complementary mode of actions that various *Bacillus* strains exhibit. A study conducted by Reuben et al. (2022) compared the effect of single strain and multi-strain probiotics in broiler chickens and found that birds supplemented with probiotics had improved growth performance, however the addition birds fed multi-strain probiotics also had lower cholesterol and glucose levels, and an increase in haemoglobin, white blood cells, and platelets, indicating the combined immunomodulatory and digestive effects were greater in multi-strain applications.

Additionally, Vimon et al. (2020) found that the combination of a *Bacillus licheniformis* and *B. subtilis* significantly improved jejunal villus height to crypt depth ratio, ileal digestibility of protein and dry matter, and had the highest content of lactic acid producing bacteria compared to the control group. Although multi-strain probiotics have been shown to improve broiler performance, health and welfare, there is limited research directly comparing various multi-strain *Bacillus* probiotics combinations, as well as comparisons against antibiotics.

Therefore, this study aimed to measure the impact of various novel *Bacillus* probiotic combinations on broiler chicken growth performance, gut morphology, nutrient digestibility, and microbial profile.

## 2 Materials and methods

### 2.1 Animal ethics statement

All the animal experiments and procedures related to animal handling and management, euthanasia, tissue sampling was carried out according to Australian code for the care and use of animals for scientific purposes (the Code). Also, all the animal experiments and relevant practices were compliant with ARRIVE guidelines and approved by Production and Companion Animal Committee of the Research Ethics and Integrity of The University of Queensland, Australia (approval certificate number: 2020/AE000235).

### 2.2 Animal experiments

#### 2.2.1 Experiment one

The 576 Ross 308 broiler chicks (mixed sex) at 1 d old involved in this experiment were purchased from Darwalla Group and transferred to the Queensland Animal Science Precinct at The University of Queensland, Gatton, Australia, for a 21-day trial. All chicks were weighed, and randomly assigned to one of 6 experimental groups in a randomised block design (Table 1). Each experimental diet was fed to 12 replicate pens (1 m × 1 m) (*n* = 72 pens) with eight birds in each (*n* = 96 birds per experimental diet). In this experiment, four combinations of feed-grade powder probiotics were incorporated into the diet at 3 × 10^5^ CFU/g feed using a 250-kg mixer (Thoroughmixer, Australia) at a concentration of 0.15% (Formulation 1, 2, 3, and 5), compared with a positive (standard diet) and a negative control (standard diet with 20 g/t tylosin) in a commercial sorghum, soybean meal, and wheat-based mash diet (Table S1). Birds receiving a probiotic treatment were administered probiotics for the entire trial period (d 0–21).

**Table 1:** Treatment groups and *Bacillus* species formulation used in Exp. 1 (6 × 10^8^ CFU/g product, 3 × 10^5^ CFU/g feed).

Treatments	Diet description	Probiotic combination
T1	Standard feed without antibiotic	Positive control
T2	T1 + tylosin (20 g/t)	Negative control
T3	T1 + F1 (0.15%)	F1 (Formulation 1) = BAM1 + BAM3 + BAM4
T4	T1 + F2 (0.15%)	F2 (Formulation 2) = BAM1 + BAM4 + BCON
T5	T1 + F3 (0.15%)	F3 (Formulation 3) = BAM1 + BAM4 + BCON + BLIC2
T6	T1 + F5 (0.15%)	F5 (Formulation 5) = BAM1 + BAM2 + BLIC2 + BSUB

BAM1, *Bacillus amyloliquefaciens* strain 1; BAM2, *B. amyloliquefaciens* strain 2; BAM3, *B. amyloliquefaciens* strain 3; BAM4, *B. amyloliquefaciens* strain 4; BLIC2, *Bacillus licheniformis* strain 2; BSUB, *Bacillus subtilis*; BCON, *Bacillus coagulans*.

All birds were provided with a commercial starter feed ad libitum (Table S1) and water for the entire three-week trial period and had ad libitum access to feed before slaughtering. Feed intake and body weight were measured weekly (end of week one, two, and three), and mortality was recorded daily. Mash feed formulation followed the starter diet composition for the entire three weeks (Table S1). At the end of week three, two broilers were randomly selected from each pen for tissue and digesta collection. Birds were sacrificed using cervical dislocation. The lighting program, temperature, and humidity followed the Ross 308 guidelines. The lighting program provided 23 h of light at 30 to 40 lx intensity and 1 h dark (less than 0.4 lx) for the first 7 d and a minimum of 4 h of darkness and a light period of 10 lx intensity after 7 d. The temperature was set at 32 °C with 40% relative humidity for the first 7 d and there was a 2 °C reduction per week after 7 d until the temperature reached 28 °C at 21 d with 40% relative humidity, as this trial concluded at 21 d.

#### 2.2.2 Experiment two

This experiment consisted of a six-week (42 d) broiler chicken performance trial. A total of 360 one-day-old Ross308 broiler chicks (mixed sex) obtained from Darwalla Group were transferred to the Queensland Animal Science Precinct at The University of Queensland, Gatton, Australia. All chicks were weighed, and randomly assigned to one of five experimental groups in a randomised block design (Table 2). Each experimental diet was fed to nine replicate pens (1 m × 1 m) (*n* = 45 pens) with eight birds in each (*n* = 72 birds per experimental diet). The feed-grade powder probiotic formulations used in this experiment were chosen based on the production performance (d 0–21) results from Exp. 1 and were incorporated into a commercial maize and soybean meal-based mash diet at 3 × 10^5^ CFU/g feed using a 250-kg mixer (Thoroughmixer, Australia) at a concentration of 500 g/t (Table S2). Birds receiving a probiotic treatment were administered probiotics for the entire trial period (d 0–42). The diet was fed according to a standard three-phase of starter, grower, and finisher, each for a two-week period. All chickens were provided with ad libitum feed and water for the entire 6 weeks and had ad libitum access to feed (Table S2). Titanium dioxide (0.2%) was mixed through the finisher feed for digestibility analysis. The remainder of the procedure was consistent with Exp. 1. The lighting program, temperature, and humidity followed the Ross 308 guidelines. The lighting program provided 23 h of light at 30 to 40 lx intensity and 1 h dark (less than 0.4 lx) for the first 7 d and a minimum of 4 h of darkness and a light period of 10 lx intensity after 7 d. The temperature was set at 32 °C with 40% relative humidity for the first 7 d and there was a 2 °C reduction per week after 7 d until the temperature reached 24 °C at 27 d with 40% relative humidity. The temperature and relative humidity were maintained until the end of the trial (Madigan-Stretton et al., 2021).

**Table 2:** Treatment groups and *Bacillus* species formulation used in Exp. 2 (6 × 10^8^ CFU/g product, 3 × 10^5^ CFU/g feed).

Treatments	Diet description	Probiotic combination
T1	Standard feed without antibiotic	Positive control
T2	T1 + tylosin (20 g/t)	Negative control
T3	T1 + F1 (500 g/t)	F1 (Formulation 1) = BAM1 + BAM3 + BAM4
T4	T1 + F4 (500 g/t)	F4 (Formulation 4) = BAM3 + BAM4 + BPUM
T5	T1 + F5 (500 g/t)	F5 (Formulation 5) = BAM1 + BAM2 + BLIC1 + BSUB

BAM1, *B. amyloliquefaciens* strain 1; BAM2, *B. amyloliquefaciens* strain 2; BAM3, *B. amyloliquefaciens* strain 3; BAM4, *B. amyloliquefaciens* strain 4; BLIC1, *B. licheniformis* strain 1; BSUB, *B. subtilis*; BPUM, *Bacillus pumilus*.

### 2.3 Gut morphology

The gut morphology methodology was reported as per (Madigan-Stretton et al., 2021). The gastrointestinal tract from the base of the gizzard down to the rectum were dissected, and 1 cm sections were cut from the mid-region of the duodenum, jejunum, and ileum, flushed with distilled water, and preserved in 10% formalin solution (*n* = 1 bird/pen; total Exp. 1, *n* = 72 birds; total Exp. 2, *n* = 45 birds). The fixed tissues were then loaded into cassettes for morphological analysis. Each fixed intestinal sample was cut into a 5-mm section and embedded in paraffin wax (Medite TES Valida embedding station). Embedded intestinal segments were cut at a thickness of 6 μm (Leica semi-automated RM2245 rotary microtome, Leica Microsystems, VIC, Melbourne, Australia) and mounted onto slides. Slides were then stained with hematoxylin and eosin, dried in the oven overnight. The slides were scanned by an Aperio ScanScope XT (Leica Microsystems, VIC, Melbourne, Australia) and analysed for the villus height, crypt depth, villus width, and the number of goblet cells. Then, the villus surface area was calculated and the villus height to crypt depth ratio was measured.

Villus height was measured from the tip of the villus to the crypt between individual villi. Crypt depth was measured from the valley between the bases of the villi to the submucosa. Villus width was calculated from the mean value of villus width at one-third and villus width at two-thirds of the height of the villus. The area between 3 villi was used from 3 cuts per sample to count the number of goblet cells. The average of the 3 measurements was then reported as the number of goblet cells per surface area.

### 2.4 Microbial profiling

#### 2.4.1 DNA extraction and sequencing

Cecal contents were collected, flash frozen in liquid nitrogen and stored at −80 °C until further analysis from 6 individuals per treatment randomly selected from different pens (*n* = 6 birds/treatment, total *n* = 36 birds). Samples were thawed and microbial DNA was extracted from 2 to 3 mg of caecal content. Samples were resuspended in tissue-lysis buffer (Promega, Australia), and underwent bead-beating treatment (using 0.1 mm and 1.0 mm zirconia beads; 3 × 60 s, 30 Hz) to ensure cell lysis. DNA extraction was conducted using the Maxwell 16 SEV cartridges (Promega, Australia) and the Maxwell 16 automated nucleic acid purification system (Promega, Australia), running in SEV (standard elution volume) mode, following the manufacturer's instructions. DNA quantification was done by using the NanoDrop instrument, following the manufacturer's instructions. The V3–V4 region of the 16S rRNA gene was amplified using specific primers (F: 5′-CCTAYGGGRBGCASCAG-3′, R: 5′-GGACTACNNGGGTATCTAAT-3′). The PCR conditions were 1 cycle of 2 min at 95 °C, 30 cycles of 20 s each at 95 °C, 55 °C for 15 s, and 72 °C for 5 min, followed by 1 cycle of 10 min at 72 °C. PCR products were sequenced, 300 bp pair-end, on Illumina MiSeq platform (AGRF, Melbourne, Australia).

#### 2.4.2 Quality filtering and sequence analysis

After demultiplexing and removing adapters, a total of 3,501,292 reads were counted, ranging from 75,602 to 129,323 reads per sample in a total of 36 samples from 6 treatments (Fig. S1). DADA2 plugin of QIIME2 was used for quality filtering and tables of representative sequences and features were generated. These sequences were assigned into 1529 unique features and classified into 7 taxonomic ranks. Taxonomy was assigned using classify-sklearn naïve Bayes taxonomy classifier against SILVA v128 database (Quast et al., 2013).

### 2.5 Digestibility analysis

After processing, the gastrointestinal tract was removed from the carcass as per Madigan-Stretton et al. (2021). The ileal content from two birds of the same pen were evacuated and pooled into a 10-mL O-ring tube and placed on ice and left in a −20 °C freezer until freeze drying occurred. The rest of the tissue was discarded. This process was repeated for all birds (*n* = 2 birds/pen for both animal experiments). The sample was weighed pre-freeze drying and post-freeze drying to calculate dry matter. To determine the ash and organic matter content, 2 g of the sample were placed in a crucible and burned in a muffle furnace for 3 h at 500 °C (AOAC, 2005), and it was calculated by the following equation:

Wt% ash = (Ashed sample – Pre-ashed sample) × 100,

Organic matter = 100 − Wt% ash.

To determine carbon, nitrogen, and sulphur content in the sample, 1 g of sample was placed into a ceramic boat in the Leco CNS 928 combustion analyzer (LECO Australia, NSW, Castle Hill, Australia) and analyzed.

The nutrient ileal digestibility was calculated using the following equation:

Nutrient apparent ileal digestibility (%) = [100 − (Ni × Md)/(Nd × Mi)] × 100,

where Ni represents a concentration of the nutrient in ileal digesta; Md represents a dietary concentration of marker; Nd represents a dietary concentration of the nutrient under the study; and Mi represents the concentration of marker in ileal digesta (Scott and Hall, 1998).

### 2.6 In vitro analyses

#### 2.6.1 Bacteria, growth conditions and preparation of cell suspension

All 9 *Bacillus* strains were obtained from the CBS-KNAW culture collection (Utrecht, Netherlands) and grown in tryptone soy broth at 37 °C with shaking at 180 rpm for 8 h. The cells were then centrifuged at 1850 × *g* for 15 min and washed 3 times with sterile phosphate buffered saline (PBS, pH 7) to remove the media. The harvested cells from each strain were subsequently resuspended and serially diluted in sterile PBS, respectively. Concentrations of the cell suspensions were defined as colony-forming unit per millilitre (CFU/mL) and were determined by a plate counting method (Latorre et al., 2016). Cell suspensions of all the strains were adjusted to 2 × 10^7^ CFU/mL prior to enzyme production measurement.

#### 2.6.2 In vitro determination of enzymes produced from the Bacillus strains

A plating assay adapted from Latorre et al. (2016) was used to investigate the abilities of *Bacillus* strains to produce extracellular amylase and protease (Latorre et al., 2016). In brief, a sterile disk was first placed on selective agar, followed by loading 50 μL cell suspension (2 × 10^7^ CFU/mL) of each *Bacillus* strain on the disk. Then, the plates were incubated at 37 °C for 36 h. After incubation, the diameters of the zone of clearance (ZC) and the diameters of the bacterial colony (BC) grown on agar plates were measured in millimetre. The relative enzyme activity (REA) equalled the value of the diameter of formed ZC divided by the diameter of the BC. All the enzyme assays were repeated in triplicate, and the average values of REA were calculated. The components of each selective agar media and evaluation methods are described below.

#### 2.6.3 Assay for amylase production

A starch agar media consisting of 10 g/L tryptone, 3 g/L potato starch, 5 g/L KH_2_PO_4_, 10 g/L yeast extract, and 15 g/L agar was prepared and autoclaved at 121 °C for 15 min, followed by cooling and pouring into sterile petri dishes for solidification. To determine the production of amylase from *Bacillus*, all the agars were flooded with Gram's iodine solution after incubation. The presence of amylase activity was indicated if the agar surrounding the BC developed a ZC (Ibrahim et al., 2012).

#### 2.6.4 Assay for protease production

To determine production of protease from bacteria, all the agars were flooded with Gram's iodine solution after incubation. The presence of protease activity was indicated if a clear zone appeared around bacteria colony (Ibrahim et al., 2012). Skim milk agar media consisting of 25 g/L agar and 25 g/L skim milk powder were prepared and autoclaved at 121 °C for 15 min. Then the agar media was kept in a water bath at 60 °C and poured quickly into sterile petri dishes to solidify. Production of protease from bacteria was determined by observing if a clear zone was developed around BC (Pailin et al., 2001).

### 2.7 Statistical analysis

The animal trial design was a randomised design with treatment effect being the only fix effect in the statistical model. Data were analysed using ANOVA in PROC GLM of SAS 9.4. Treatments were compared using the Tukey test, and a threshold of 0.05 was considered for significant *P*-values.

For the gut morphology, nutrient digestibility, and *Bacillus* enzyme production, a general linear model was fitted to the data which was then analysed using ANOVA. Upon significant effect from diet, post-hoc Tukey Honest Significant Difference testing was conducted to find the groupings of the diets. The assumptions of the model were checked using a Fitted value versus Residual plot as well as Quantile–Quantile plot of the Residuals against a Normal Distribution. All models met the assumptions. The data was split by type of animal and gut position. The analysis was conducted in the R Statistical Programming language using the emmeans package and multcomp for a compact letter display summary of the Tukey honestly significance difference tests (Hothorn et al., 2008; Lenth, 2023).

For the microbial profiling, data from QIIME2 was imported into R Statistical Software (RStudio Team, 2020) for further downstream analysis and visualisation. QIIME2 artifacts were imported and transformed into a phyloseq file using the qiime2R package (Bisanz, 2018). Alpha diversity, rarefactions, beta diversity, heatmaps of taxa and spearman correlations were performed and visualized using several packages including phyloseq (McMurdie and Holmes, 2013), microbiome utilities (Shetty and Lahti, 2022), microViz, and metaMisc (Mikryukov, 2018). Wilcoxon signed-rank test was used to observe the significant differences (*P* ≤ 0.05) between treatments. Beta-diversity was plotted using principal coordinate analysis (PCoA) and the distance between treatments was calculated using the Bray–Curtis method. Heatmaps were used to visualize the differences in terms of relative abundances of top taxa between treatments. Spearman correlation was performed between taxa abundances and average daily gain (ADG), average daily feed intakes (ADFI), and feed conversion ratio (FCR).

## 3 Results and discussion

### 3.1 Performance parameters

Experiment one was designed as a preliminary experiment to test the efficacy of 4 strain combinations (formulation 1 [F1]: 3 strains *Bacillus amyloliquefaciens*, F2: *Bacillus coagulans* and 2 strains *B. amyloliquefaciens*, F3: *B. coagulans, B. licheniformis* and 2 strains *B. amyloliquefaciens*, and F5: *B. subtilis, B. licheniformis* and 2 strains *B. amyloliquefaciens*). In addition, two control groups of T1: positive control (PC) which was a feed without antibiotics, and T2: negative control (NC) feed with antibiotic tylosin were included. In Exp. 1, F1 (T3) compared to F3 (T5) showed significantly (*P* = 0.027) higher body weight at d 21 and higher average daily gain d 1 to 21 (Table 3). T3 had a significantly better FCR compared to F2 (T4) at d 14 (*P* = 0.038). The accumulated FCR for the whole 21 d for T3 tended to be the lowest compared to other treatments (*P* = 0.068). The European broiler index (EBI) was significantly higher in T3 than T5 (*P* = 0.023) and numerically higher than all other treatment groups. In addition, there was a trend (*P* = 0.085) affecting the mortality rates where T3 displayed the lowest rate with no mortality (0%). The addition of T3 to the broiler diet improved the performance and decrease the mortality indicating a better health in the flock. T3 tested probiotic F1 consisting of a combination of three *B. amyloliquefaciens* strains. The effect of spore-based probiotics on broiler production performance has been well documented (Bilal et al., 2021; Mazanko et al., 2022; Popov et al., 2021; Zhang et al., 2021). *B. amyloliquefaciens* has been used successfully in broiler and layer diets to improve production performance, intestinal morphology, and microbial profile due to its production of antimicrobial compounds, enzymes, and good colonisation abilities in the broiler gastrointestinal tract (Gharib-Naseri et al., 2021). This was also reflected in the enzyme assays presented in Table 4, as BAM2 (*B. amyloliquefaciens* strain 2), BAM3 (*B. amyloliquefaciens* strain 3), and BAM4 (*B. amyloliquefaciens* strain 4) produced significantly higher amylase and protease enzymes compared to other strains tested. The production of enzymes aid in improving feed digestibility as the nutrients are more accessible to the broiler, which may explain the improvement in production performance in T3. T4 contained *B. coagulans* and resulted in poorer ADFI (0–7 d) and FCR (0–14 d) compared to T3 (Table 3). Similarly, Zhang et al. (2021) demonstrated that the addition of *B. coagulans* did not impact ADFI or FCR, but it increased BW together with altering immunological parameters in broilers chickens. In contrast, in an earlier study Wang and Gu (2010) demonstrated that *B. coagulans* improved the final body weight associated with an increased protease and amylase activities consistent with the tested strain. However, whilst the amylase and protease assay demonstrated a significantly increased enzyme production compared to other strains, it was significantly less than BAM3 and BAM4 (Table 4). One of the limitations of this study is that the optimal performance objectives set by the Ross 308 guideline were not met due to unknown circumstances. Broilers consuming T3 showed improved performance compared to the control group. Putting together all the in vitro and in vivo data the *Bacillus* combination was selected and used in a 42-d trial to examine its effect using a standard commercial broiler production period (Table 3, Table 4).

**Table 3:** Effect of different *Bacillus* strains on performance parameters of broiler chickens (Exp. 1, *n* = 576).

Item	Experimental diet 1						SEM	*P*-value
	T1	T2	T3	T4	T5	T6		
BW, g								
d 0	41.8	41.3	42.1	42.4	42.1	42.8	0.39	0.142
d 7	163.2	159.7	164.8	162.2	162.8	164.8	2.84	0.812
d 14	420.5	423.7	436.1	419.6	415.3	428.8	7.81	0.477
d 21	808.1^ab^	834.8^ab^	847.0^a^	814.4^ab^	787.4^b^	843.4^ab^	14.07	0.027
ADG, g								
d 0–7	17.3	16.9	17.5	17.1	17.2	17.4	0.38	0.891
d 7–14	36.7	37.7	38.7	36.7	36.0	37.7	0.78	0.201
d 14–21	55.3	58.7	58.6	56.4	53.1	59.2	1.47	0.053
d 0–21	36.4^ab^	37.7^ab^	38.3^a^	36.7^ab^	35.4^b^	38.1^ab^	0.66	0.041
ADFI, g								
d 0–7	26.2^ab^	25.6^ab^	23.8^b^	28.8^a^	26.6^ab^	26.1^ab^	1.03	0.013
d 7–14	70.4	63.7	58.0	76.7	67.1	64.3	4.60	0.099
d 14–21	82.9	81.6	85.9	80.6	76.9	78.1	2.99	0.326
d 0–21	60.3	58.0	55.9	62.4	57.8	57.0	1.77	0.122
FCR, g/g								
d 0–7	1.52^ab^	1.53^ab^	1.36^b^	1.69^a^	1.55^ab^	1.51^ab^	0.079	0.046
d 7–14	1.92^ab^	1.70^ab^	1.49^b^	2.10^a^	1.88^ab^	1.72^ab^	0.142	0.038
d 14–21	1.50	1.38	1.46	1.43	1.45	1.32	0.049	0.143
d 0–21	1.66	1.54	1.46	1.70	1.64	1.50	0.060	0.068
CV, %								
d 7	11.8	14.8	11.2	13.1	14.0	11.6	1.21	0.230
d 14	13.0	13.9	14.1	14.7	16.2	21.1	1.28	0.311
d 21	12.6	11.7	12.4	13.1	13.7	9.0	1.17	0.091
Mortality, %	2.1	4.2	0.0	2.1	7.3	4.2	2.06	0.085
EBI	219^ab^	234^ab^	264^a^	215^ab^	207^b^	243^ab^	12.6	0.023

BW, body weight; ADG, average daily gain; ADFI, average daily feed intake; FCR, feed conversion ratio; CV, coefficient of variation (indication of growth uniformity calculated as a ratio of the standard deviation to the mean); EBI, European broiler index, which is (ADG × survival rate)/(FCR × 10).

^a, b^ Within a row, means with different letters are significantly different at *P* < 0.05 level.

1 T1, positive control (PC) of a standard commercial feed without antibiotics; T2, negative control (NC) of standard feed with antibiotic tylosin, at 20 g/t; T3, PC + formulation 1 which included 3 strains of *B. amyloliquefaciens* (0.15%); T4, PC + formulation 2 which included *B. coagulans* and 2 strains of *B. amyloliquefaciens* (0.15%); T5, PC + formulation 3 which included *B. coagulans*, *B. licheniformis* and 2 strains of *B. amyloliquefaciens* (0.15%); T6, PC + formulation 5 which included *B. subtilis*, *B. licheniformis* and 2 strains of *B. amyloliquefaciens* (0.15%).

**Table 4:** Relative enzyme activity (REA) values^1^ produced by *Bacillus* strains.

Strain^2^	Amylase, REA	Protease, REA
BAM1	1.15^d^	1.89^d^
BPUM	1.00^e^	1.79^d^
BAM3	1.36^c^	2.25^b^
BAM4	1.46^b^	2.28^b^
BLIC1	1.04^e^	1.83^d^
BCON	1.37^bc^	2.53^a^
BAM2	1.70^a^	2.64^a^
BLIC2	1.21^d^	1.23^e^
BSUB	1.74^a^	2.06^c^
SEM	0.027	0.044
*P*-value	0.001	<0.001

BAM1, *B. amyloliquefaciens* strain 1; BAM2, *B. amyloliquefaciens* strain 2; BAM3, *B. amyloliquefaciens* strain 3; BAM4, *B. amyloliquefaciens* strain 4; BLIC1, *B. licheniformis* strain 1; BLIC2, *B. licheniformis* strain 2; BSUB, *B. subtilis*; BCON, *B. coagulans*; BPUM, *Bacillus pumilus*.

^a-e^ Within a column, means with different letters are significantly different at *P* < 0.05 level.

1 Relative enzyme activity values were calculated dividing the diameter of area of clearance by the diameter of the *Bacillus* colony.

2 All *Bacillus* strains were tested by triplicate.

In Exp. 2, birds from T4 had a higher BW than T5 on d 28 (*P* = 0.014) (Table 5). Similarly, the ADG of T4 from d 14 to 28 was significantly higher than T2 and T5 (*P* = 0.003). In terms of FCR, T4 performed significantly (*P* < 0.001) better than T2 and T5 from d 14 to 28. This shifted from d 28 to 42, whereby T1, T2, and T5 had a significantly lower FCR than T3 (*P* < 0.001, Table 5).

**Table 5:** Effect of different *Bacillus* strains on performance parameters of broiler chickens (Exp. 2, *n* = 360).

Item	Experimental diet^1^					SEM	*P*-value
	T1	T2	T3	T4	T5		
BW, g							
d 0	40.0	39.9	40.8	40.1	40.1	0.36	0.419
d 14	425.2	436.8	434.6	426.1	424.8	7.50	0.679
d 28	1391.0^ab^	1383.6^ab^	1445.8^ab^	1457.1^a^	1360.7^b^	22.09	0.014
d 42	2652.0	2704.7	2693.5	2750.1	2615.2	48.84	0.362
ADG, g							
d 0–14	27.5	28.4	28.1	27.3	27.5	0.57	0.622
d 14–28	69.0^ab^	66.7^b^	72.2^ab^	73.6^a^	65.9^b^	1.57	0.003
d 28–42	90.1	94.4	89.1	92.4	89.6	2.10	0.369
d 0–42	62.2	63.4	63.2	64.5	60.5	1.29	0.269
ADFI, g							
d 0–14	34.4	38.0	34.9	34.9	35.6	1.44	0.425
d 14–28	103.2	106.5	107.3	107.9	109.7	1.86	0.171
d 28–42	158.3	161.0	165.4	165.3	157.3	3.49	0.338
d 0–42	98.6	101.8	102.5	102.7	100.9	1.74	0.461
FCR, g/g							
d 0–14	1.22	1.36	1.21	1.25	1.26	0.076	0.649
d 14–28	1.49^bc^	1.59^abc^	1.49^bc^	1.46^c^	1.68^a^	0.027	<0.001
d 28–42	1.75^b^	1.69^b^	1.84^a^	1.78^ab^	1.74^b^	0.022	<0.001
d 0–42	1.59^b^	1.61^ab^	1.62^ab^	1.59^ab^	1.67^a^	0.019	0.030
CV, %							
d 28	9.7	11.2	8.5	9.7	12.7	1.04	0.067
d 35	9.7	11.4	8.5	9.9	12.8	1.08	0.071
d 42	10.3	11.9	9.5	10.2	13.4	1.29	0.227
Mortality, %	1.4	1.4	0.0	4.2	1.4	1.42	0.350
EBI	387.6	390.2	389.2	387.9	360.4	13.15	0.452

BW, body weight; ADG, average daily gain; ADFI, average daily feed intake; FCR, feed conversion ratio; CV, coefficient of variation (indication of growth uniformity calculated as a ratio of the standard deviation to the mean); EBI, European broiler index, which is (ADG × survival rate)/(FCR × 10).

^a, b^ Within a row, means with different letters are significantly different at *P* < 0.05 level.

1 T1 = positive control (PC) of a standard commercial feed without antibiotics; T2 = negative control (NC) of standard feed with antibiotic tylosin, at 20 g/t; T3 = PC + F1 which included 3 strains of *B. amyloliquefaciens* (500 g/t); T4 = PC + F4 which included *Bacillus pumilus* and 2 strains of *B. amyloliquefaciens* (500 g/t); T5 = PC + F5 which included *B. subtilis*, *B. licheniformis*, and 2 strains of *B. amyloliquefaciens* (500 g/t).

**Fig. 1:**
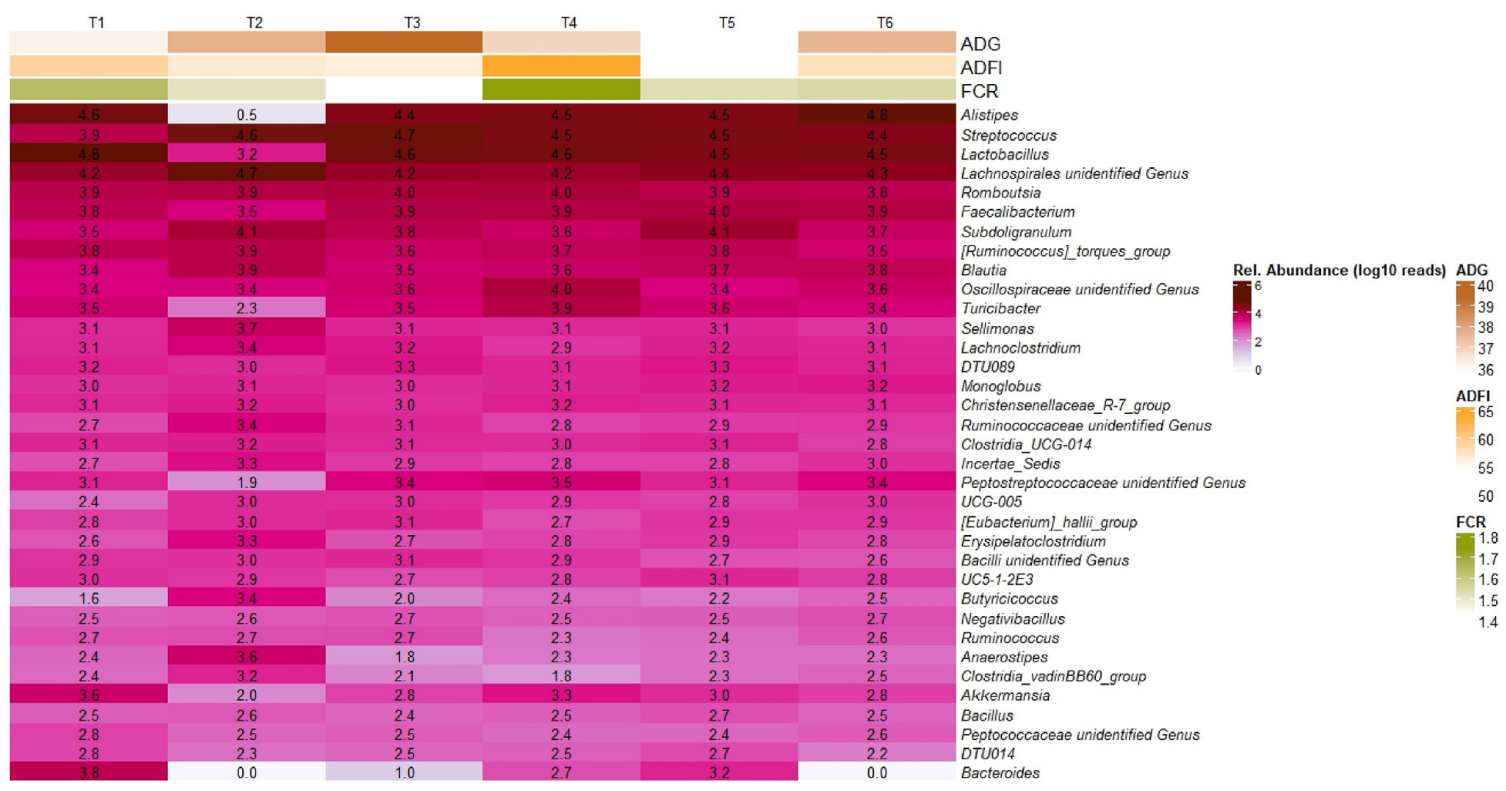
Most abundant genera in each treatment in Exp. 1. Relative abundance (reads) was transformed to log_10_. ADG = average daily gain; ADFI = average daily feed intake; FCR = feed conversion ratio. T1 = positive control (PC) of a standard commercial feed without antibiotics; T2 = negative control (NC) of standard feed with antibiotic tylosin, at 20 g/t; T3 = PC + F1 which included 3 strains of *B. amyloliquefaciens* (0.15%); T4 = PC + F2 which included *B. coagulans* and 2 strains of *B. amyloliquefaciens* (0.15%); T5 = PC + F3 which included *B. coagulans*, *B. licheniformis* and 2 strains of *B. amyloliquefaciens* (0.15%); T6 = PC + F5 which included *B. subtilis*, *B. licheniformis* and 2 strains of *B. amyloliquefaciens* (0.15%).

**Fig. 2:**
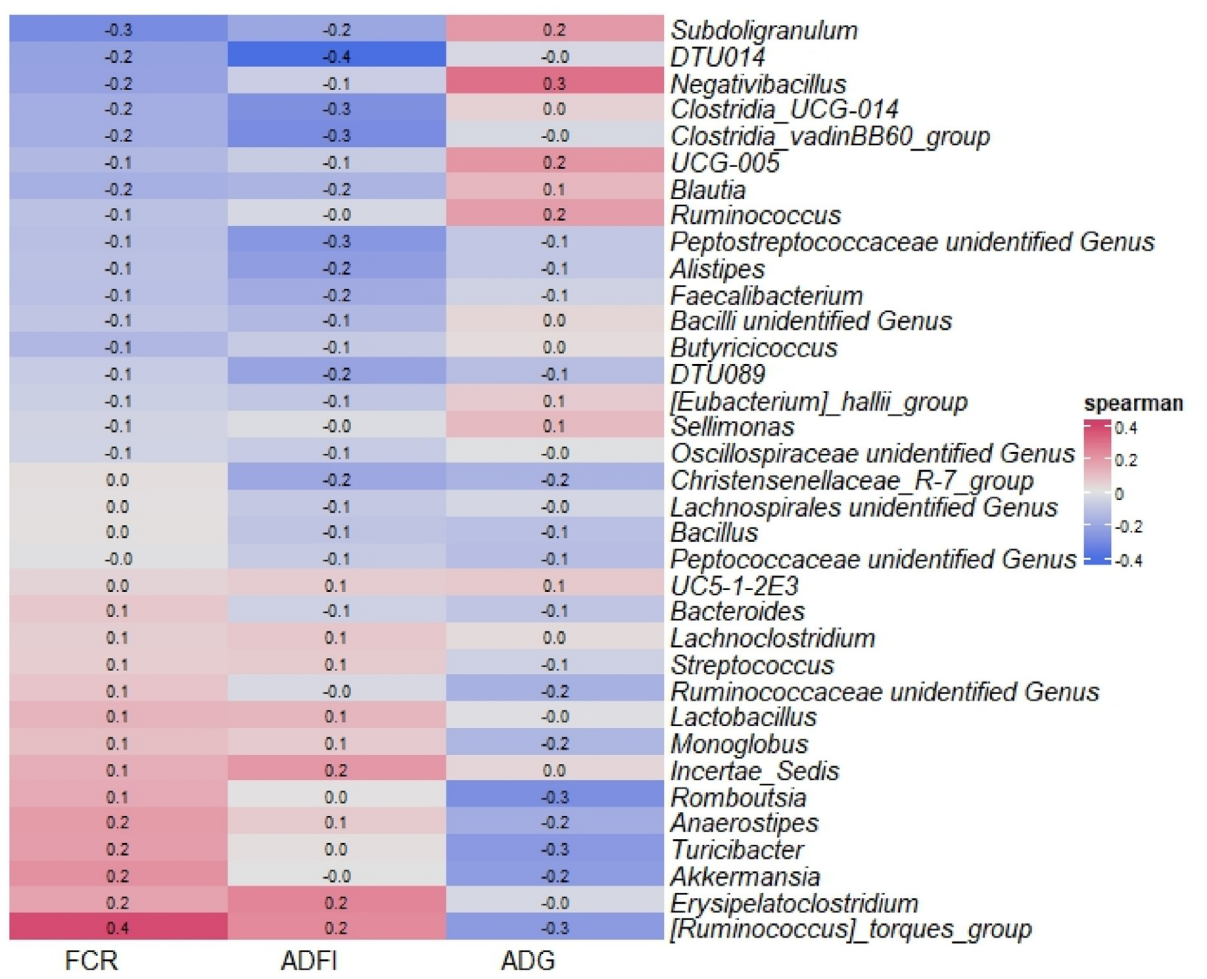
Spearman correlation between most taxonomic abundances at genus level and growth performance factors in Exp. 1 including average daily gain (ADG), average daily feed intakes (ADFI), and feed conversion ratio (FCR).

Previous literature showed similar results in production performance, with improved BW and FCR when including *Bacillus pumilus* in the diet (Bilal et al., 2021; Bonos et al., 2021). The higher production performance in *Bacillus* supplemented diets seem to reflect the ability to modulate the intestinal environment, such as decreasing the pH and increasing the number of lactic acid producing bacteria, which renders the gut as an unsuitable environment for pathogenic organisms (Bonos et al., 2021; Deniz et al., 2011). The microbial profiling from Exp. 1 presented in section 3.4 confirmed the increased presence of *Lactobacillus* and other genera that improve the growth performance and health of broilers in diets supplemented with probiotics (Fig. 1, Fig. 2). Lactic acid produced by *Lactobacillus* has antipathogenic effect and improve nutrient availability resulting in better performance (García-Hernández et al., 2016; Natsir et al., 2010). Production of enzymes such as protease and amylase by the *Bacillus* spp. also contributes to increased nutrient digestibility and improved performance parameter in broilers (Park et al., 2020; Riaz et al., 2020).

### 3.2 Gut morphology

The results showed no significant differences between treatment groups in terms of gut morphology measurements in the first experiment (Table 6). However, Exp. 2 revealed that T5 had significantly longer villus height than T2, and a significantly longer crypt depth in jejunum than the remaining treatment groups (*P* = 0.025 and *P* = 0.040, respectively) (Table 7). Aliakbarpour et al. (2012) found similar results when investigating the effects of *B. subtilis* on growth performance and gut morphology in broiler chickens, as the villus height and crypt depth were not statistically significant amongst treatment groups. However, there are variable results within the existing literature as to the effect of probiotics on the gut morphology, that the addition of *Bacillus* in broiler diets significantly improved the villus height and crypt depth compared to the control (Bonos et al., 2021; Wealleans et al., 2017). It is possible that probiotics may have had their positive impacts on other aspects of gut development or other part of the gastrointestinal tract such as cecum that have not been analysed in this study.

**Table 6:** Effect of different *Bacillus* strains on the gut morphology of 21-d-old broiler chickens (Exp. 1).

Parameter	Experimental diet^1^						SEM	*P*-value
	T1	T2	T3	T4	T5	T6		
Duodenum, μm								
Villus height	1028.1	1074.0	1109.2	1114.8	1187.8	952.0	128.35	0.573
Crypt depth	106.4	81.3	104.9	105.2	119.5	104.8	12.14	0.466
Villus width	510.8	432.0	542.3	488.1	462.3	462.9	56.94	0.504
Jejunum, μm								
Villus height	904.5	846.3	942.9	944.4	925.4	953.3	69.75	0.743
Crypt depth	77.7	62.8	69.3	70.4	72.5	66.2	8.73	0.646
Villus width	440.0	401.4	384.2	454.3	445.4	392.4	42.39	0.403
Ileum, μm								
Villus height	613.4	646.1	658.2	653.7	709.3	691.7	43.74	0.285
Crypt depth	62.7	60.3	49.7	62.8	71.9	56.7	9.04	0.253
Villus width	376.7	414.3	372.8	450.9	434.8	434.1	44.17	0.374

1 T1 = positive control (PC) of a standard commercial feed without antibiotics; T2 = negative control (NC) of standard feed with antibiotic tylosin, at 20 g/tonne; T3 = PC + F1 which included 3 strains of *B. amyloliquefaciens* (0.15%); T4 = PC + F2 which included *B. coagulans* and 2 strains of *B. amyloliquefaciens* (0.15%); T5 = PC + F3 which included *B. coagulans*, *B. licheniformis* and 2 strains of *B. amyloliquefaciens* (0.15%); T6 = PC + F5 which included *B. subtilis*, *B. licheniformis* and 2 strains of *B. amyloliquefaciens* (0.15%).

**Table 7:** Effect of different *Bacillus* strains on the gut morphology of 42-d-old broiler chickens (Exp. 2).

Parameter	Experimental diet^1^					SEM	*P*-value
	T1	T2	T3	T4	T5		
Duodenum, μm							
Villus height	2056.0	1405.0	1809.0	1876.0	1552.0	146.10	0.280
Crypt depth	50.9	65.4	84.9	76.3	78.7	18.31	0.476
Villus width	526.0	785.0	795.0	970.0	850.0	165.26	0.292
Jejunum, μm							
Villus height	947.0^ab^	773.0^b^	1084.0^ab^	1019.0^ab^	1176.0^a^	103.31	0.025
Crypt depth	64.7^b^	71.8^b^	71.2^b^	82.9^b^	121.4^a^	12.94	0.040
Villus width	616.0	539.0	531.0	668.0	622.0	116.86	0.250
Ileum, μm							
Villus height	611.0	580.0	596.0	600.0	607.0	68.87	0.996
Crypt depth	79.2	75.4	68.6	91.3	82.3	8.63	0.439
Villus width	496.0	569.0	572.0	540.0	540.0	77.90	0.969

^a, b^ Within a row, means with different letters are significantly different at *P* < 0.05 level.

1 T1 = positive control (PC) of a standard commercial feed without antibiotics; T2 = negative control (NC) of standard feed with antibiotic tylosin, at 20 g/t; T3 = PC + F1 which included 3 strains of *B. amyloliquefaciens* (500 g/t); T4 = PC + F4 which included *Bacillus pumilus* and 2 strains of *B. amyloliquefaciens* (500 g/t); T5 = PC + F5 which included *B. subtilis*, *B. licheniformis*, and 2 strains of *B. amyloliquefaciens* (500 g/t).

### 3.3 *Bacillus* enzyme production

All nine *Bacillus* strains were cultured to evaluate their secretion of amylase and protease (Table 4). Extracellular enzyme activities were observed among all the strains. By comparing their REA values between the two enzymes, all strains exhibited higher protease activity than amylase activity. Strain BAM2, BAM3, BAM4, BCON (*B. coagulans*), and BSUB (*B. subtilis*) exerted strong protease productivity with a REA value larger than 2.0. Protease secretion was significantly superior in BAM2 and BCON, exceeding the activity of the remaining seven strains, whereas BLIC2 (*B. licheniformis* strain 2) produced the lowest amount of protease (*P* < 0.001). In the case of amylase activity, strain BSUB produced significantly more than the remaining eight strains (*P* = 0.010). In contrast, strain BPUM (*B. pumilus*) did not demonstrate a larger ZC than its BC, indicating no secretion of amylase. BAM1 and BLIC1 also produced minimal amylase.

Poultry feed often includes ingredients, such as wheat, barley, rye, and sorghum, which can contain high levels of non-starch polysaccharides (NSP) and other antinutritional factors not digested by endogenous pancreatic enzymes (Knudsen, 2014). To address this issue, the feed industry has been incorporating exogenous enzymes, like carbohydrases, into animal diets, as they can alleviate the negative effects of NSP and other antinutritional factors and enhance nutrient availability and utilization (Slominski, 2011). Probiotics, such as *Bacillus* genus, can secrete fibre-degrading enzymes that complement endogenous enzyme activity (Casula and Cutting, 2002; Latorre et al., 2016; Tran et al., 2023). Some researchers referred to probiotics as “live enzyme factories”, emphasizing their capacity to produce enzymes like amylase, protease, and lipase (Zaghari et al., 2020). This enzymatic aid has been reported to improve nutrient digestibility in poultry (Latorre et al., 2016). In addition, unlike feed enzymes, which must demonstrate resilience in terms of thermal stability, pH tolerance, and resistance to proteolytic degradation, *Bacillus* probiotics offer an approach by synthesizing extracellular enzymes on-site in the host organism's digestive tract (Danilova and Sharipova, 2020).

### 3.4 Microbial profiling

**Fig. 3:**
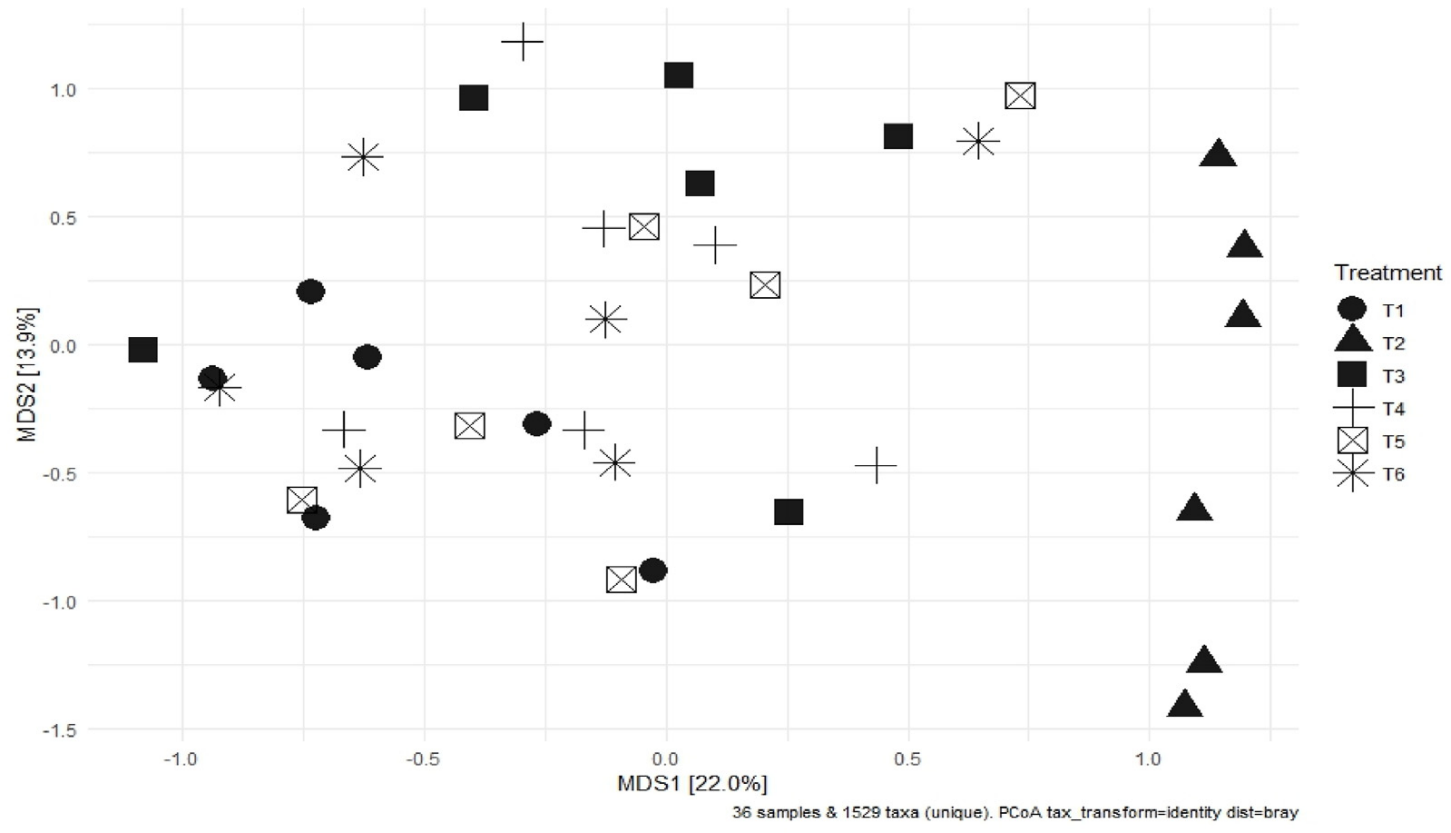
The impact of treatments in Exp. 1 on beta-diversity in which two metric multidimensional scaling (MDS) are shown. MSD1 explaining 22.0% of dissimilarity and MDS2 explaining 13.9% of dissimilarity. T1 = positive control (PC) of a standard commercial feed without antibiotics; T2 = negative control (NC) of standard feed with antibiotic tylosin, at 20 g/tonne; T3 = PC + F1 which included 3 strains of *B. amyloliquefaciens* (0.15%); T4 = PC + F2 which included *B. coagulans* and 2 strains of *B. amyloliquefaciens* (0.15%); T5 = PC + F3 which included *B. coagulans*, *B. licheniformis* and 2 strains of *B. amyloliquefaciens* (0.15%); T6 = PC + F5 which included *B. subtilis*, *B. licheniformis* and 2 strains of *B. amyloliquefaciens* (0.15%).

Beta-diversity measures the dissimilarity in terms of taxonomic abundances. PCoA plot was used to estimate the beta-diversity based on the Bray–Curtis distance (Fig. 3). Only T2 clustered at the right side of the plot indicating that the antibiotic treatment had a clear impact in the microbiome profile. Alpha-diversity indices including Chao 1, Shannon, and Simpson are shown in Fig. S2.

**Fig. 4:**
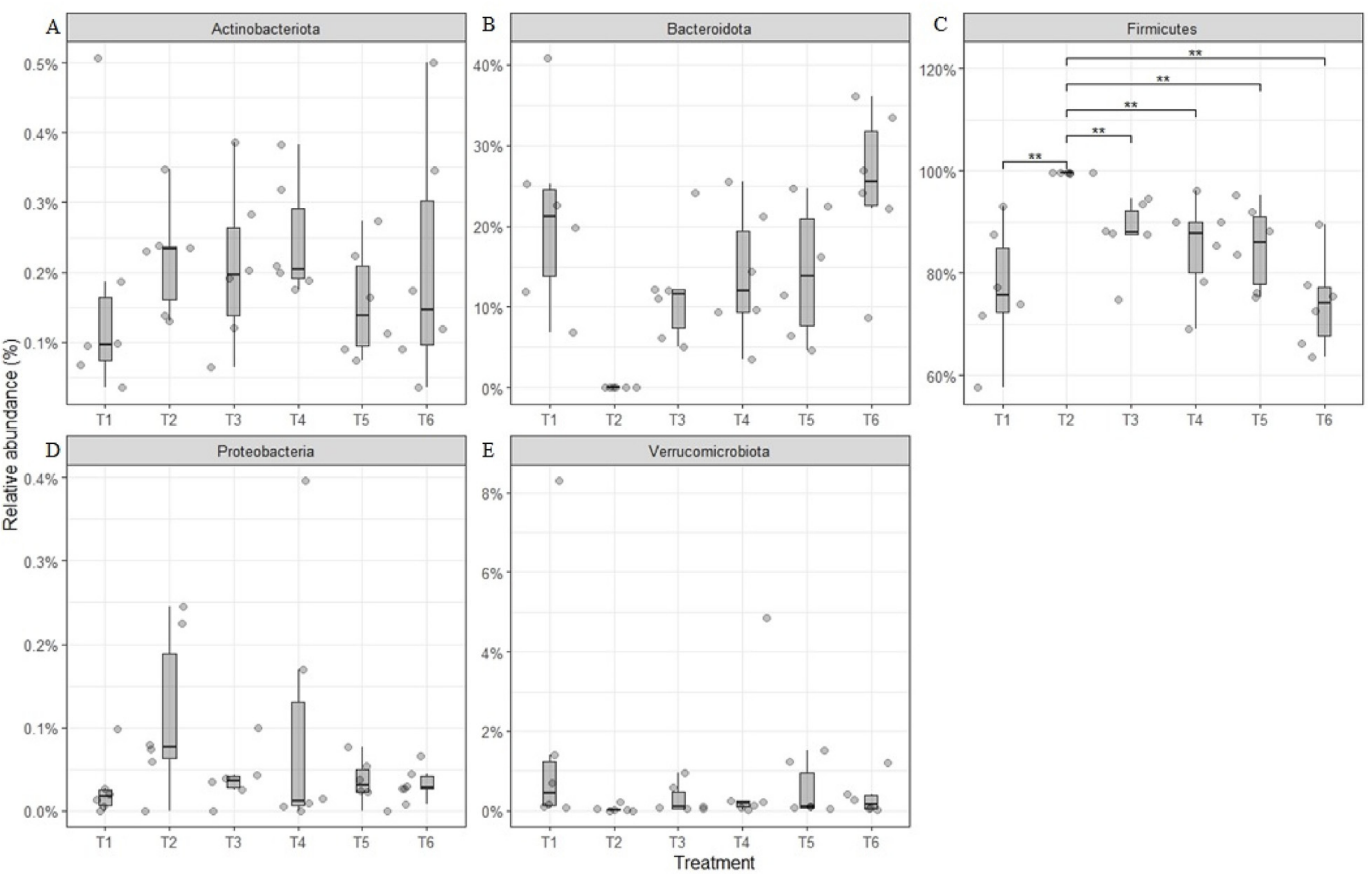
Relative abundances of phyla in each treatment in Exp. 1. (A) Phylum Actinobacteriota, (B) phylum Bacteroidota, (C) phylum Firmicutes, (D) phylum Proteobacteria, and (E) phylum Verrucomicrobiota. T1 = positive control (PC) of a standard commercial feed without antibiotics; T2 = negative control (NC) of standard feed with antibiotic tylosin, at 20 g/t; T3 = PC + F1 which included 3 strains of *B. amyloliquefaciens* (0.15%); T4 = PC + F2 which included *B. coagulans* and 2 strains of *B. amyloliquefaciens* (0.15%); T5 = PC + F3 which included *B. coagulans*, *B. licheniformis* and 2 strains of *B. amyloliquefaciens* (0.15%); T6 = PC + F5 which included *B. subtilis*, *B. licheniformis* and 2 strains of *B. amyloliquefaciens* (0.15%). ∗∗ Indicates significant differences at *P* < 0.01.

At the phylum level, 90% of the microbiota belongs to Firmicutes and Bacteroidota. The proportion of those phyla was significantly affected in the group receiving antibiotic (T2) (Fig. 4). Firmicutes accounted for approximately 99% of total relative abundance in T2 compared with the remaining treatments (*P* = 0.001), while in the case of Bacteroidota phylum was the opposite, with a strong reduction in T2. This difference suggested the strong effect of antibiotics on Bacteroidota while Firmicutes might possess antibiotic-resistant properties, as reported by Singh et al. (2013) with the use of penicillin in broilers. In addition, strong antibiotic resistance of Firmicutes phyla compared to Bacteroidota has been reported (Chatzikonstantinou et al., 2021).

The core microbes of the microbiota were selected for visualisation based on their relative abundance. Details of their relative abundance at genus level in each treatment was provided in Table S3. Overall, the gut microbiota in samples was found to be strongly influenced by antibiotic treatments when applying ANOVA test. However, this statistical analysis might undermine the impacts of probiotics treatments. To effectively illustrate the shift of microbiota between treatments, the mean of relative abundance at genus level in each treatment was used to construct a heatmap (Fig. 1). Spearman correlation between genera abundance with growth performance are shown in Fig. 2.

At the genus level, *Streptococcus*, a common commensal bacterium in the gut consists of both beneficial and pathogenic species (Cisek and Binek, 2014) is favoured with the dietary addition of the probiotics as well with the addition of the antibiotic (Fig. 1). Spearman correlation showed *Streptococcus*, is highly correlated with ADG (Fig. 2). *Negativibacillus* was increased in T3, T5, and T6 (combination of *B. subtilis, B. licheniformis* and 2 strains of *B. amyloliquefaciens*) and positively correlated with high ADG (Fig. 1, Fig. 2). This genus was shown to negatively correlate with FCR and ADFI (Fig. 2). T4 recorded a high relative abundance of *Turicibacter* genus, which was often associated with *Bacillus* treatment for chickens (Memon et al., 2022) (Fig. 1). *Turicibacter* suggested to positively correlate with butyrate production, an indicator for gut health (Zhong et al., 2015). Additionally, it also often correlated with high FCR (Siegerstetter et al., 2017), similar to our results (Fig. 2).

All probiotic combinations seemed to reduce the abundance of *Bacteroides* and *Akkermansia,* especially in T3 and T6 (Fig. 1). *Bacteroidetes* genus is also a common commensal bacterium with the ability to ferment and degrade indigestible fibres in the cecum (Polansky et al., 2015). However, some species in the *Bacteroidetes* genus can be opportunistic pathogens (Wexler, 2007). The *Akkermansia* genus was found to be related with mucin-degradation and usually negatively correlated with body weight in animals (Everard et al., 2013). In this study, a negative correlation between relative abundances of *Akkermansia* and ADG was also observed (Fig. 2). These results suggest the probiotic treatments suppressed the genera associated with low ADG.

The antibiotic in the feed dramatically reduced both *Alistipes* and *Lactobacillus* groups (Fig. 1), which were recognised as healthy genera for the host gut. *Alistipes* was shown to correlate with high body weight in broiler chickens (Farkas et al., 2022). However, our results indicated a negative correlation between those genera and ADG (Fig. 2). These contradictions could be explained by the differences among species which belong to the *Alistipes* genus (Parker et al., 2020). *Lactobacillus* appeared to have correlation with ADFI and FCR (Fig. 2). This genus was also shown to enhance the feed intake, immune respond in gut, and weight gain (Yan et al., 2017).

Overall, while antibiotics could considerably disturb the balance of microbiota and may reduce beneficial and normal commensal bacteria, administrating *Bacillus* probiotic could stimulate a healthy microbial profile and promote the growth performance. Nevertheless, the information acquired at genus level might not be sufficient to provide clear explanations. Therefore, extent research can be done to have more insight on the change of microbiota and their correlation with other growth factors.

### 3.5 Nutrient digestibility

Table 8, Table 9 summarise the results on nutrient digestibility for Exps. 1 and 2, respectively. T3 showed the highest N content, which was significantly higher than the negative control (T2) and T4 (*P* = 0.009) (Table 8). T1, T5, and T6 also had significantly higher N than T2 (*P* = 0.009). Similar results were also seen in the crude protein (CP) content with T2 being the lowest compared to other treatments (*P* = 0.009). As demonstrated in Table 4, the probiotic strains used in these formulations produced amylase and protease as part of their metabolic process. The production of these enzymes by the various *Bacillus* species enables nutritional benefits to the animal due to the improvement in accessible protein, which enables increased muscle deposition. High bioavailability of proteins creates modifications in myogenic regulatory factor expression, including satellite activation, myogenic determination and muscle cell differentiation (Park et al., 2020). Moreover, probiotics like *B. licheniformis* supplementation improved protein content and essential amino acids, as well as meat quality and chemical elements deposition in breast muscles of broilers (Duskaev et al., 2020). A similar study by Zaghari et al. (2020) has shown that adding dietary *B. subtilis* and *B. licheniformis* improved apparent digestibility of CP and metabolizable energy compared to the control group (Zaghari et al., 2020). Thus, *Bacillus* spp. potential in producing digestive enzymes, such as amylase, protease and lipase can enhance the digestion and absorption of carbohydrate, proteins and lipids, and contribute to improving feed efficiency. *Bacillus* spp. was also known to produce cellulase and xylanase (Kogut and Arsenault, 2016). Protease produced by *Bacillus* spp. can aid the digestion of proteins into smaller peptides and amino acids, which is consistent with better N and CP digestibility (Table 8). In Exp. 1, T1 revealed a significantly higher Al content than the other treatment groups (*P* = 0.016) (Table 8). Mn was significantly higher in T1 than in T3, T4, and T6, and T6 was significantly lower than T5 and T1 (*P* < 0.05). Moisture content, total ash content, carbon, and remaining macro and micro minerals were not significantly different between treatment groups (Table 8). Experiment two revealed a significantly higher Ca and P digestibility in T2 compared to T3, while Na digestibility was significantly higher in T1 compared to T3 (Table 9). This is consistent with the performance data from Exp. 2 (Table 5), whereby T1 and T2 has a significantly lower FCR than F5, suggesting that optimal utilisation of these nutrient is required for ideal performance.

**Table 8:** Effect of different *Bacillus* strains on the nutrient apparent ileal digestibility of broiler chickens at d 21 (Exp. 1).

Parameter	Experimental diet^1^						SEM	*P*-value
	T1	T2	T3	T4	T5	T6		
Moisture content, wt%	79.2	79.8	79.4	79.8	79.2	79.4	0.61	0.879
Total ash, wt%	12.0	11.5	10.8	11.0	11.4	10.6	0.66	0.366
Carbon, wt%	41.1	41.4	41.5	41.0	41.7	42.3	0.86	0.684
N, wt%	3.0^ab^	2.8^c^	3.2^a^	3.0^bc^	3.0^ab^	3.0^ab^	0.10	0.009
Crude protein, wt%	19.3^ab^	17.6^c^	20.3^a^	18.8^bc^	19.3^ab^	19.1^ab^	0.63	0.009
Al, mg/kg	520^a^	394^b^	390^b^	389^b^	381^b^	348^b^	49.7	0.016
B, mg/kg	60.2	44.4	49.8	73.0	58.3	77.9	18.23	0.433
Ca, wt%	2.6	2.2	2.1	2.3	2.5	2.0	0.31	0.403
Cu, mg/kg	85.4	145.6	63.1	67.3	72.6	67.5	37.26	0.266
Fe, mg/kg	880.2	810.4	838.5	770.8	885.4	748.0	78.59	0.390
K, wt%	0.5	0.7	0.6	0.6	0.6	0.5	0.07	0.054
Mg, wt%	0.7	0.7	0.7	0.6	0.7	0.7	0.05	0.291
Mn, mg/kg	359.0^a^	316.0^abc^	298.0^bc^	294.0^bc^	332.0^ab^	262.0^c^	29.35	0.029
Na, wt%	0.7	0.8	0.8	0.9	0.8	0.8	0.09	0.226
P, wt%	1.3	1.1	1.1	1.2	1.3	1.0	0.13	0.133
S, wt%	0.4	0.4	0.4	0.5	0.4	0.4	0.08	0.690
Zn, mg/kg	426.0	378.8	377.0	374.0	604.2	307.8	96.73	0.064
Ti, mg/kg	2978.0	2190.0	1883.0	2057.0	2083.0	1630.0	751.29	0.571

^a, b, c^ Within a row, means with different letters are significantly different at *P* < 0.05 level.

1 T1 = positive control (PC) of a standard commercial feed without antibiotics; T2 = negative control (NC) of standard feed with antibiotic tylosin, at 20 g/tonne; T3 = PC + F1 which included 3 strains of *B. amyloliquefaciens* (0.15%); T4 = PC + F2 which included *B. coagulans* and 2 strains of *B. amyloliquefaciens* (0.15%); T5 = PC + F3 which included *B. coagulans*, *B. licheniformis* and 2 strains of *B. amyloliquefaciens* (0.15%); T6 = PC + F5 which included *B. subtilis*, *B. licheniformis* and 2 strains of *B. amyloliquefaciens* (0.15%).

**Table 9:** Effect of different *Bacillus* strains on the nutrient apparent ileal digestibility of broiler chickens at d 42 (Exp. 2).

Parameter^1^	Experimental diet^2^					SEM	*P*-value
	T1	T2	T3	T4	T5		
Moisture content, wt%	79.4	79.5	78.6	78.2	78.9	0.21	0.631
Al, mg/kg	156.5	130.1	131.1	128.6	134.9	4.99	0.597
B, mg/kg	93.1	54.2	51.3	62.2	61.6	4.48	0.201
Ca, wt%	5.43^ab^	5.64^a^	3.73^b^	4.46^ab^	4.47^ab^	0.168	0.012
Cu, mg/kg	80.5	76.1	76.5	74.1	78.3	1.77	0.974
Fe, mg/kg	1075.5	680.2	554.4	520.2	631.9	60.34	0.317
K, wt%	1.1	0.9	1.3	1.0	1.2	0.04	0.189
Mg, wt%	0.51^ab^	0.47^b^	0.63^a^	0.55^ab^	0.62^ab^	0.022	0.004
Mn, mg/kg	343.2	391.6	324.4	347.1	352.9	10.41	0.653
Na, wt%	0.81^a^	0.75^ab^	0.52^b^	0.64^ab^	0.67^ab^	0.020	0.009
P, wt%	1.87^ab^	2.02^a^	1.34^b^	1.65^ab^	1.59^ab^	0.051	0.020
S, wt%	0.4	0.3	0.3	0.3	0.3	0.01	0.190
Zn, mg/kg	409.8	413.8	400.1	425.9	440.6	7.66	0.278
Ti, mg/kg	2.3	2.8	2.8	2.4	3.0	0.20	0.565

^a, b^ Within a row, means with different letters are significantly different at *P* < 0.05 level.

1 Due to an unfortunate incident in the laboratory, some of the samples were lost and the four parameters “total ash”, “carbon”, “N”, and “crude protein” were lost.

2 T1 = positive control (PC) of a standard commercial feed without antibiotics; T2 = negative control (NC) of standard feed with antibiotic tylosin, at 20 g/t; T3 = PC + F1 which included 3 strains of *B. amyloliquefaciens* (500 g/t); T4 = PC + F4 which included *Bacillus pumilus* and 2 strains of *B. amyloliquefaciens* (500 g/t); T5 = PC + F5 which included *B. subtilis*, *B. licheniformis*, and 2 strains of *B. amyloliquefaciens* (500 g/t).

## 4 Conclusion

Dietary supplementation of two *B. amyloliquefaciens* strains significantly improved nutrient digestibility and (lower) mortality rates in broiler chickens. The *B*. *amyloliquefaciens* strains were able to produce higher levels of amylase and protease that could boost the digestive capability of the broilers. In addition, the probiotic supplementation reduced microbial genera associated with low weight gain compared to the controls. Probiotic supplementation did not affect the histological parameters studied.

## Author contributions

**Jacoba Bromfield**: conceptualization, data curation, funding acquisition, formal analysis, investigation, methodology, project administration, supervision, validation, visualization, writing - original draft, writing - review & editing. **Shahram Niknafs**: conceptualization, data curation, formal analysis, investigation, methodology, project administration, supervision, validation, visualization, writing - review & editing. **Xiaojing Chen**: Conceptualization, funding acquisition, project administration, resources, visualization, writing - original draft, writing - review & editing. **Juhani von Hellens**: conceptualization, project administration, resources, funding acquisition. **Darwin Horyanto, Lei Yu**: project administration, resources, data curation. **Viet Hai Tran**: data curation, formal analysis, writing - original draft and visualisation. **Marta Navarro**: data curation, formal analysis, investigation, methodology, project administration, writing - review & editing. **Baode Sun**: formal analysis, methodology, writing - review & editing. **Eugeni Roura**: methodology, formal analysis, project administration resources, writing - review & editing.

## Declaration of competing interest

We declare that we have no financial and personal relationships with other people or organizations that can inappropriately influence our work, and there is no professional or other personal interest of any nature or kind in any product, service and/or company that could be construed as influencing the content of this paper.
