# Supplementary material for: The evaluation of next-generation probiotics on broiler growth performance, gut morphology, gut microbiome, nutrient digestibility, in addition to enzyme production of Bacillus spp. in vitro
Source: Anim Nutr. 2024 Apr 6;18:133–44. doi: 10.1016/j.aninu.2024.03.013 (PMC11388156; doi:10.1016/j.aninu.2024.03.013)
Supplement: Multimedia component 1 [file mmc1.docx]

**Supplementary materials**

**Table S1** Ingredient and chemical composition of the starter feed used in the animal Exp. 1 (as fed basis, %).

| **Ingredients** | **Content** |
| --- | --- |
| Barley 10% hammered | 10.00 |
| Sorghum hammered | 35.83 |
| Wheat 11% hammered | 20.00 |
| Soybean meal | 21.10 |
| Blood meal | 1.05 |
| Meat bone meal | 10.00 |
| Vegetable oil mix | 1.00 |
| Salt - micro-mix | 0.16 |
| Lysine HCl - micro | 0.22 |
| DL-Methionine | 0.23 |
| Feed enzyme mix | 0.02 |
| Mycotoxin degrader | 0.07 |
| Betaine | 0.10 |
| Vitamin premix^1^ | 0.10 |
| Mineral premix^2^ | 0.10 |
| **Nutrient composition** |  |
| Dry matter | 89.51 |
| Protein | 22.98 |
| Crude fibre | 2.73 |
| AME, MJ/kg | 12.96 |
| Arginine | 1.40 |
| Leucine | 1.85 |
| Isoleucine | 0.85 |
| Lysine | 1.30 |
| Methionine | 0.61 |
| Threonine | 0.81 |
| Tryptophan | 0.26 |
| Valine | 1.07 |
| Methionine + Cysteine | 0.95 |
| Calcium | 1.36 |
| Phosphorus | 1.00 |
| Available Phosphorus | 0.56 |
| Calcium/Phosphorus | 1.36 |
| Sodium | 0.16 |
| Potassium | 0.72 |
| Chloride | 0.24 |
| Magnesium | 0.18 |
| Salt | 0.16 |
| Copper, mg/kg | 10.00 |
| Selenium, mg/kg | 0.30 |
| Vitamin A, IU/kg | 10 |
| Vitamin D3, IU/kg | 3000 |
| Vitamin E, mg/kg | 40.00 |
| Biotin, mg/kg | 0.15 |
| Choline, mg/kg | 2835.16 |
| Fat | 4.37 |
| Linoleic acid | 1.05 |

**^1^**Vitamin premix: vitamin A, 20 MIU; vitamin D3, 5 MIU; vitamin E, 60 g; vitamin K, 4 g; nicotinic acid (vitamin B), 60 g; pantothenic acid (vitamin B5), 20 g; folic acid, 2.4 g; riboflavin (vitamin B2), 8 g; cyanocobalamin (vitamin B12), 0.03 g; biotin, 0.2 g; pyridoxin (vitamin B6), 8 g; thiamine (vitamin B1), 2.4 g.

^2^ Mineral premix: copper, 20 g; cobalt, 0.4 g; molybdenum: 1.0 g; iodine: 2 g; selenium: 0.4 g; iron: 60 g; zinc: 140 g; manganese: 140 g.

**Table S2** Ingredients and chemical composition of the basal diet used in the animal Exp. 2 (as fed basis, %).

| **Ingredients** | **Starter** | **Grower** | **Finisher** |
| --- | --- | --- | --- |
| Maize 9.0 hammered / roll | 63.68 | 68.54 | 70.35 |
| Soy hull pellets | - | - | 2.50 |
| Soyabean meal 46% | 24.85 | 20.8 | 17.30 |
| Meat and bone meal | 8.60 | 7.60 | 6.90 |
| Vegetable oil - mix | 1.00 | 1.00 | 1.00 |
| Limestone | 0.75 | 1.00 | 1.00 |
| Sodium bicarbonate | 0.05 | 0.05 | - |
| Salt - micro | 0.20 | 0.20 | 0.25 |
| Lysine HCL | 0.247 | 0.22 | 0.14 |
| DL-Methionine | - | 0.20 | 0.18 |
| Threonine |  |  | 0.06 |
| Feed enzyme mix | 0.02 | 0.02 | 0.02 |
| Mycotoxin degrader | 0.08 | 0.06 | 0.04 |
| Betaine | 0.1 | 0.1 | 0.05 |
| Vitamin premix | 0.1 | 0.1 | 0.1 |
| Mineral premix | 0.1 | 0.1 | 0.1 |
| **Chemical composition** |  |  |  |
| Dry matter | 88.62 | 88.50 | 88.45 |
| Protein | 22.03 | 20.06 | 18.50 |
| Crude fibre | 2.54 | 2.45 | 3.20 |
| AME, MJ/kg | 12.85 | 13.00 | 12.81 |
| Arginine | 1.40 | 1.25 | 1.13 |
| Leucine | 1.84 | 1.73 | 1.62 |
| Isoleucine | 0.85 | 0.77 | 0.70 |
| Lysine | 1.30 | 1.15 | 1.00 |
| Methionine | 0.61 | 0.56 | 0.50 |
| Threonine | 0.81 | 0.73 | 0.73 |
| Tryptophan | 0.23 | 0.21 | 0.19 |
| Valine | 1.00 | 0.91 | 0.84 |
| Methionine + Cysteine | 0.95 | 0.88 | 0.80 |
| Calcium | 1.47 | 1.43 | 1.35 |
| Phosphorus | 0.90 | 0.83 | 0.78 |
| Available phosphorous | 0.50 | 0.46 | 0.43 |
| Calcium/Phosphorous | 1.62 | 1.71 | 1.73 |
| Sodium | 0.17 | 0.17 | 0.17 |
| Potassium | 0.69 | 0.61 | 0.58 |
| Chloride | 0.22 | 0.21 | 0.23 |
| Magnesium | 0.21 | 0.20 | 0.20 |
| Salt | 0.20 | 0.20 | 0.25 |
| Copper, mg/kg | 10.0 | 10.0 | 10.0 |
| Selenium, mg/kg | 0.30 | 0.30 | 0.30 |
| Vitamin A, IU/kg | 10.0 | 10.0 | 10.0 |
| Vitamin D3, IU/kg | 3000 | 3000 | 3000 |
| Vitamin E, mg/kg | 40.0 | 40.0 | 40.0 |
| Biotin, mg/kg | 0.15 | 0.15 | 0.15 |
| Choline, mg/kg | 2774.60 | 2663.90 | 1782.00 |
| Fat | 4.70 | 4.72 | 4.71 |
| Linoleic acid | 1.61 | 1.66 | 1.69 |
|  |  |  |  |

**^1^**Vitamin premix: vitamin A, 16.667 MIU; vitamin D3, 4.167 MIU; vitamin E, 50 g; vitamin K, 3.333 g; nicotinic acid (vitamin B3), 50 g; pantothenic acid (vitamin B5), 16.667 g; folic acid, 2 g; riboflavin (vitamin B2), 6 g; cyanocobalamin (vitamin B12), 0.025 g; biotin,0.167 g; pyridoxin (vitamin B6), 6.666 g; thiamine (vitamin B1), 2 g.

^2^ Mineral premix: copper, 16.66 g; cobalt, 0.333 g; molybdenum, 0.833 g; iodine, 1.666 g; selenium, 0.333 g; iron, 50 g; zinc, 116.667 g; manganese, 116.667 g.

**Table S3** The most abundant genera (reads count) among treatments.

| Genera | Experimental diets | | | | | | SEM | *P*-value |
| --- | --- | --- | --- | --- | --- | --- | --- | --- |
|  | T1 | T2 | T3 | T4 | T5 | T6 |  |  |
| *Alistipes* | 6315.5^a^ | 0.3^b^ | 4280.0^ab^ | 5113.5^ab^ | 4776.3^ab^ | 9767.8^a^ | 571.28 | <0.001 |
| *Bacteroides* | 1021.6 | 0.0 | 1.3 | 83.8 | 236.0 | 0.0 | 130.77 | 0.126 |
| *Oscillospiraceae unidentified Genus* | 391.0 | 435.5 | 616.6 | 1677.0 | 403.3 | 738.8 | 237.92 | 0.519 |
| *UCG-005* | 37.8 | 180.8 | 176 | 125.1 | 105.5 | 157.6 | 30.78 | 0.688 |
| *Monoglobus* | 163.8 | 214.3 | 182.8 | 207.0 | 262 | 289.5 | 27.96 | 0.715 |
| *Peptococcaceae unidentified Genus* | 107.1 | 47.5 | 56.8 | 42.1 | 46 | 61.5 | 12.28 | 0.578 |
| *Christensenellaceae_R-7_group* | 203.5 | 271.1 | 173.8 | 248.1 | 208.8 | 197.1 | 20.87 | 0.708 |
| *Akkermansia* | 630.5 | 16.8 | 106.3 | 316.8 | 185.6 | 114.5 | 99.46 | 0.442 |
| *DTU014* | 100.6 | 36.5 | 48.6 | 55.5 | 90.8 | 25.3 | 12.67 | 0.369 |
| *Lactobacillus* | 10434.3 | 277.0 | 7079.6 | 6124.8 | 4781.1 | 5149.5 | 1198.15 | 0.209 |
| *Bacilli unidentified Genus* | 144.6 | 158.6 | 208.3 | 139.0 | 74.5 | 69.0 | 32.07 | 0.740 |
| *Streptococcus* | 1212.0 | 6910.0 | 7737.1 | 5181.6 | 5015 | 4196.5 | 762.83 | 0.143 |
| *Turicibacter* | 567.0 | 31.3 | 537.3 | 1388.5 | 599.3 | 459.3 | 143.88 | 0.128 |
| *Bacillus* | 56.1 | 67.5 | 46.5 | 56.6 | 80.8 | 49.1 | 11.82 | 0.944 |
| *Erysipelatoclostridium* | 72.5 | 356.8 | 77.8 | 100.8 | 128.1 | 95.1 | 31.78 | 0.065 |
| *Romboutsia* | 1226.1 | 1465.8 | 1646 | 1716.5 | 1201.6 | 1093.3 | 239.51 | 0.947 |
| *Peptostreptococcaceae unidentified Genus* | 230.5 | 13.5 | 416.3 | 531.0 | 232.5 | 394.8 | 59.27 | 0.123 |
| *Clostridia_vadinBB60_group* | 40.0^ab^ | 235.5^a^ | 19.8^b^ | 10.6^b^ | 35.5^b^ | 49.3^ab^ | 20.51 | 0.014 |
| *[Eubacterium]_hallii_group* | 109.6 | 183.6 | 218.0 | 83.3 | 148.1 | 139.0 | 28.61 | 0.713 |
| *Negativibacillus* | 53.6 | 65.1 | 75.1 | 53.5 | 58.3 | 83.0 | 10.61 | 0.931 |
| *Ruminococcaceae unidentified Genus* | 92.1 | 425.5 | 209.0 | 107.1 | 119.6 | 131.6 | 37.22 | 0.068 |
| *Ruminococcus* | 75.3 | 84.5 | 89.1 | 32.1 | 38.5 | 69.1 | 11.10 | 0.474 |
| *DTU089* | 249.6 | 183.3 | 305.6 | 212.3 | 298.1 | 230.8 | 23.97 | 0.554 |
| *Incertae_Sedis* | 80.5^b^ | 338.1^a^ | 126.0^ab^ | 106.0^b^ | 102.5^b^ | 162.8^ab^ | 22.56 | 0.012 |
| *Faecalibacterium* | 1031.8 | 469.8 | 1402.1 | 1475.1 | 1569.8 | 1436.5 | 270.99 | 0.794 |
| *Subdoligranulum* | 512.8 | 1895.8 | 1140.6 | 725.3 | 2213.5 | 774.3 | 227.49 | 0.133 |
| *Butyricicoccus* | 7.1^b^ | 384.0^a^ | 18.3^b^ | 41.1^ab^ | 29.3^ab^ | 48.6^ab^ | 37.51 | 0.025 |
| *Anaerostipes* | 46.1 | 633.0 | 10.3 | 29.5 | 35.3 | 35.0 | 80.78 | 0.132 |
| *Lachnospiraceae unidentified Genus* | 2562.5^b^ | 8696.5^a^ | 2636.1^b^ | 2592.1^b^ | 3845.8^b^ | 3436.3^b^ | 347.77 | <0.001 |
| *Sellimonas* | 192.5^b^ | 838.8^a^ | 208.6^b^ | 194.8^b^ | 217.1^b^ | 152.1^b^ | 48.02 | <0.001 |
| *Clostridia_UCG-014* | 213.1 | 260.5 | 190.1 | 185.5 | 232.1 | 113.6 | 28.88 | 0.696 |
| *Lachnoclostridium* | 198.6 | 452.6 | 292.0 | 140.5 | 245.1 | 205.8 | 32.77 | 0.079 |
| *Blautia* | 418.6 | 1210.3 | 563.6 | 688.1 | 898.0 | 955.1 | 98.04 | 0.158 |
| *UC5-1-2E3* | 162.8 | 131.5 | 83.0 | 110.1 | 206.5 | 116.1 | 19.04 | 0.403 |
| *[Ruminococcus]_torques_group* | 1121.1 | 1204.3 | 713.6 | 916.1 | 1000.8 | 581.1 | 127.89 | 0.629 |

T1 = positive control (PC) of a standard commercial feed without antibiotics; T2 = negative control (NC) of standard feed with antibiotic tylosin, at 20 g/t; T3 = PC + F1 which included 3 strains of *Bacillus amyloliquefaciens* (0.15%); T4 = PC + F2 which included *Bacillus coagulans* and 2 strains of *Bacillus amyloliquefaciens* (0.15%); T5 = PC + F3 which included *Bacillus coagulans*, *Bacillus licheniformis* and 2 strains of *Bacillus amyloliquefaciens* (0.15%); T6 = PC + F5 which included *Bacillus subtilis*, *Bacillus licheniformis* and 2 strains of *Bacillus amyloliquefaciens* (0.15%).

^a, b^ Within a row, means with different letters are significantly different at *P <* 0.05 level.


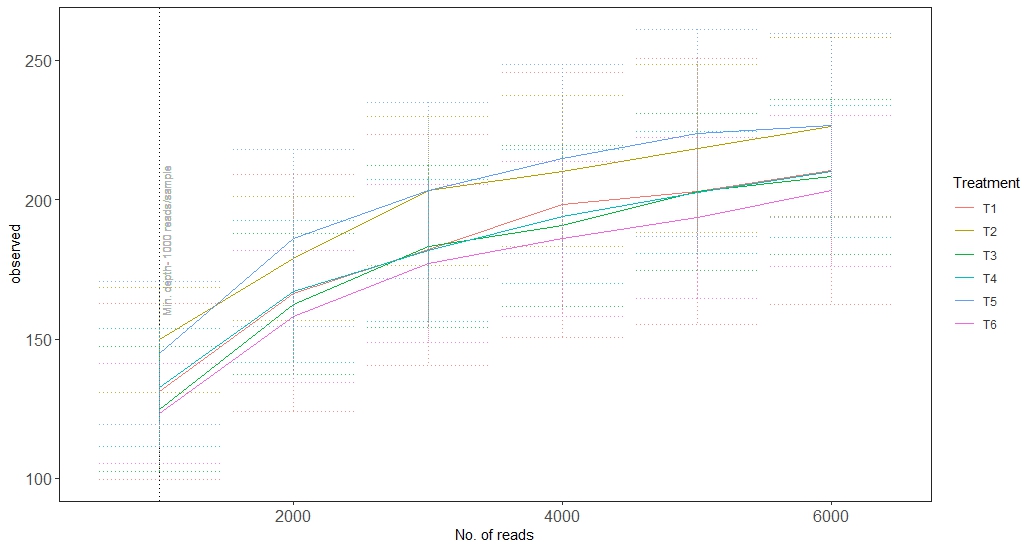


**Fig. S1.** Rarefaction of treatments on observed OTUs. T1 = positive control (PC) of a standard commercial feed without antibiotics; T2 = negative control (NC) of standard feed with antibiotic Tylosin, at 20 g/t; T3 = PC + F1 which included 3 strains of *Bacillus amyloliquefaciens* (0.15%); T4 = PC + F2 which included *Bacillus coagulans* and 2 strains of *Bacillus amyloliquefaciens* (0.15%); T5 = PC + F3 which included *Bacillus coagulans*, *Bacillus licheniformis* and 2 strains of *Bacillus amyloliquefaciens* (0.15%); T6 = PC + F5 which included *Bacillus subtilis*, *Bacillus licheniformis* and 2 strains of *Bacillus amyloliquefaciens* (0.15%).


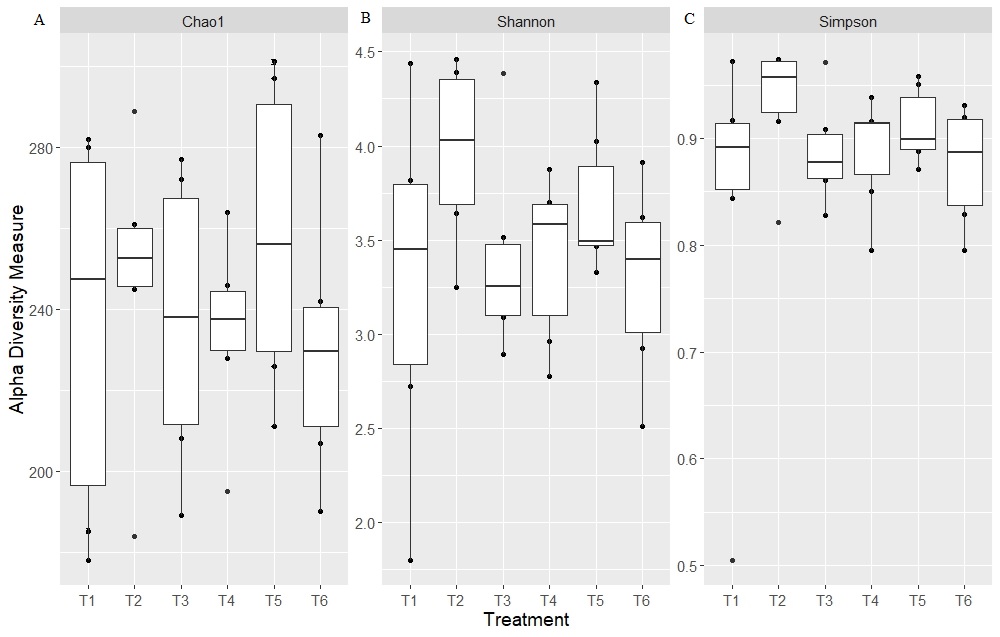


**Fig. S2.** Effect of different treatments on alpha-diversity indices included (A) Chao1 index, (B) Shannon index, and (C) Simpson index. T1 = positive control (PC) of a standard commercial feed without antibiotics; T2 = negative control (NC) of standard feed with antibiotic Tylosin, at 20 g/t; T3 = PC + F1 which included 3 strains of *Bacillus amyloliquefaciens* (0.15%); T4 = PC + F2 which included *Bacillus coagulans* and 2 strains of *Bacillus amyloliquefaciens* (0.15%); T5 = PC + F3 which included *Bacillus coagulans*, *Bacillus licheniformis* and 2 strains of *Bacillus amyloliquefaciens* (0.15%); T6 = PC + F5 which included *Bacillus subtilis*, *Bacillus licheniformis* and 2 strains of *Bacillus amyloliquefaciens* (0.15%).
